# Correction: Ethnic variation and the relevance of homozygous RNF 213 p.R4810.K variant in the phenotype of Indian Moya moya disease

**DOI:** 10.1371/journal.pone.0249469

**Published:** 2021-03-25

**Authors:** The PLOS ONE Staff

The fifth author’s name appears incorrectly. The correct name is: P. N. Sylaja. The correct citation is: K. A, Shafeeque CM, Sudhir JB, Banerjee M, Sylaja PN (2020) Ethnic variation and the relevance of homozygous RNF 213 p.R4810.K variant in the phenotype of Indian Moya moya disease. PLoS ONE 15(12): e0243925. https://doi.org/10.1371/journal.pone.0243925

The publisher apologizes for the error.
