# Supplementary material for: Ethnic variation and the relevance of homozygous RNF 213 p.R4810.K variant in the phenotype of Indian Moya moya disease
Source: PLoS One. 2020 Dec 28;15(12):e0243925. doi: 10.1371/journal.pone.0243925 (PMC7769475; doi:10.1371/journal.pone.0243925)
Supplement: Attachment — Submitted filename: Review 08.09.2020 ARL.docx [file pone.0243925.s001.docx]

Corresponding author: Sylaja P.N., MD., DM (FRCP), (FESO)

The aim of the study is clear and describe the Polymorphisms in Ring Finger protein RNF213 suspected to Moyamoya disease (MMD, especially the risk homozygous genotype AA with the SNP rs112735431 (p.R4810K) where arginine changes to lysine. In the introduction, the MMD disease is described as a rare heterogeneous disease and it is a serious condition where blood vessels in the brain become narrowed over time and this can result in the risk of blood clots and the risk of having stroke or seizure. The study is very relevant.

The study investigates MMD in Indian patients. This genotypic characterization of Indian MMD has not been studied systematically until date according to the article, and it also makes the study relevant.

The case-control study involved 65 Moyamoya disease (MMD) patients, 75 parents, 1 son of the affected person, and 120 controls. 5 patients out of 65 carry the risk genotype AA. None of the healthy controls had the genotype AA and had no typical MMD symptoms, but the controls were not screened.

22 SNPs were selected. In table1 there is a list of the SNP’s but it seems that there is only 21.

The methods of Sanger Sequencing was performed. More information about the companies of the kits/primers which has been used will be relevant to mention in the article.

Table 3. I need as a reader some explanation about the selection of the SNPs from table 1 compared to table 3. In table 3 the controls are mention but now it is 104 + 1 compare to 120 controls from the abstract - how should that be understood. How you calculated the odds ratio? The confidence interval 3.11-626 for rs112735431 is not consistent with the CI in table 3.

Table 4 just minor things about the seizure and stroke 42 (93) 3(3?).

The discussion is very interesting about the incidence of MMD in populations and that it should be notest that cases of homozygous p.R4810K in unaffected control populations also are reported and that environmental factors may have influence in the phenotype variation.

The study concludes that there is a particular risk of SNP rs112735431 (p.R4810K) with the genotype AA concerning MMD in this case report study of Indian patients but also in relation to predict the phenotype of those patients. All patients with the SNP rs112735431 (p.R4810K) AA genotype had according to this study early age of onset.

A very fine section about the limitation of this study with the opportunity to continue working within this very interesting field of polymorphisms in Ring Finger protein RNF213 in MMD diseases.

The conclusion from the reviewer. Very interesting and relevant study about polymorphism in Ring Finger protein RNF213 and MMD. The tables should be more clear as already mention (SNP, controls) but I looking forward to following this interesting field.
