# Supplementary material for: Ethnic variation and the relevance of homozygous RNF 213 p.R4810.K variant in the phenotype of Indian Moya moya disease
Source: PLoS One. 2020 Dec 28;15(12):e0243925. doi: 10.1371/journal.pone.0243925 (PMC7769475; doi:10.1371/journal.pone.0243925)
Supplement: Attachment — Submitted filename: Response to Comments.docx [file pone.0243925.s003.docx]

**Response to Comments**

Dear Editor/ Reviewer,

Thanks for your valuable comments,

1. In the revised manuscript we have revised the ‘Statistical Analysis’ section with explicit explanation of all statistical tests and functional assessments of the SNPs used.
2. We have also included the details on RegulomeDB rank and also presented it in the ‘table 1’ for better clarity.
3. Grammatical errors have been corrected and highlighted in the manuscript.
4. Edits have been made in the paragraph explaining the ‘limitations of the study’.

# **Statistical Analysis**

Demographic and phenotypic observations were analysed using SPSS 22.0 (SPSS Inc., Chicago, IL) and the continuous variables were presented as means ± standard deviation and categorical variables were presented as proportions. Genotype and allele frequencies were computed using the Graph Pad Prism 5.01, (Graph Pad software Inc. San Diego, CA, USA). A p-value of <0.05 was considered significant in all observations. The test of association for rs112735431 RNF 213 risk AA genotype with various phenotype variables such as clinical symptoms, age at onset, unilateral/bilateral, distribution of vasculopathy, imaging characteristics, and Suzuki classification, was done using Chi Square test. Linkage Disequilibrium (LD) plots for controls and patients were generated using Haploview 4.2 (broad.mit.edu/mpg/haploview/). LD plots in cases and control display how non-random association of alleles at two or more loci may differ in cases and control. Functional prediction of the associated SNPs was assessed in-silico using regulomeDB (regulome.stanford.edu), Genotype-Tissue Expression (GTEx) portal (www.gtexportal.org/), HaploReg (www.broadinstitute.org/mammals/haploreg). The functional significance of the SNPs was checked for the RegulomeDB rank to assess the possible regulatory effect. RegulomDB rank is derived from RegulomeDB database that annotates SNPs with known and predicted regulatory elements in the intergenic regions of the *H. sapiens* genome[13]. Lower scores indicate increasing evidence for a variant to be located in a functional region. Category 1 variants have equivalents in other categories with the additional requirement of expression quantitative trait loci (eQTL) information. RegulomeDB score is computed based on the integration of multiple high-throughput datasets. GTex portal was assessed for possible gene expression of the allelic variants. GTEx database allow the users to view and download computed eQTL results and provide a controlled access system for de-identified individual-level genotype, expression, and clinical data.  HaploReg was also used, which is a tool for exploring annotations of the noncoding genome at variants on haplotype blocks, such as candidate regulatory SNPs at disease-associated loci. Interpreted data is presented only with reference to associated risk SNP.
